# Molecular evolution of genes in avian genomes

**DOI:** 10.1186/gb-2010-11-6-r68

**Published:** 2010-06-23

**Authors:** Kiwoong Nam, Carina Mugal, Benoit Nabholz, Holger Schielzeth, Jochen BW Wolf, Niclas Backström, Axel Künstner, Christopher N Balakrishnan, Andreas Heger, Chris P Ponting, David F Clayton, Hans Ellegren

**Affiliations:** 1 Department of Evolutionary Biology, Evolutionary Biology Centre, Uppsala University, Norbyvägen 18D, Uppsala, S-752 36, Sweden; 2 Institute for Genomic Biology, University of Illinois, 601 S. Goodwin Avenue, Urbana, IL 61801, USA; 3 MRC Functional Genomics Unit, Department of Physiology, Anatomy and Genetics, University of Oxford, South Parks Road, Oxford, OX1 3QX, UK

## Abstract

**Background:**

Obtaining a draft genome sequence of the zebra finch (*Taeniopygia guttata*), the second bird genome to be sequenced, provides the necessary resource for whole-genome comparative analysis of gene sequence evolution in a non-mammalian vertebrate lineage. To analyze basic molecular evolutionary processes during avian evolution, and to contrast these with the situation in mammals, we aligned the protein-coding sequences of 8,384 1:1 orthologs of chicken, zebra finch, a lizard and three mammalian species.

**Results:**

We found clear differences in the substitution rate at fourfold degenerate sites, being lowest in the ancestral bird lineage, intermediate in the chicken lineage and highest in the zebra finch lineage, possibly reflecting differences in generation time. We identified positively selected and/or rapidly evolving genes in avian lineages and found an over-representation of several functional classes, including anion transporter activity, calcium ion binding, cell adhesion and microtubule cytoskeleton.

**Conclusions:**

Focusing specifically on genes of neurological interest and genes differentially expressed in the unique vocal control nuclei of the songbird brain, we find a number of positively selected genes, including synaptic receptors. We found no evidence that selection for beneficial alleles is more efficient in regions of high recombination; in fact, there was a weak yet significant negative correlation between ω and recombination rate, which is in the direction predicted by the Hill-Robertson effect if slightly deleterious mutations contribute to protein evolution. These findings set the stage for studies of functional genetics of avian genes.

## Background

There are nearly 10,000 known species of birds and many of these have been instrumental in studies of general aspects of behavior, ecology and evolution. Such basic knowledge on life history and natural history will become an important resource for studies aiming at elucidating the genetic background to phenotypic evolution in natural bird populations [1]. There have already been some attempts in this direction, including the demonstration that the calmodulin pathway is involved in the evolution of the spectacular differences in beak morphology among Darwin's finches [2,3] and the critical role of *MC1R *governing variation in plumage color in several bird species [4].

At the genomic level, birds have attracted the attention of biologists for several reasons. First, compared to other vertebrates, avian genomes are compact, with estimated DNA content typically in the range of 1.0 to 1.5 Gb, about half to one-third of the amount of DNA found in most mammals [5]. It seems clear that this is mainly due to a relatively low activity of transposable elements in birds [6]. Second, the avian karyotype is largely conserved [7] and is characterized by a high degree of conserved synteny. In contrast to mammals, avian chromosomes show significant variation in size, with the karyotype of many species containing five to ten large chromosomes ('macrochromosomes') that are comparable in size to small to medium-sized human chromosomes, and a large number of very small chromosomes (<20 Mb) referred to as microchromosomes. Third, birds have female heterogamety, with the Z and W sex chromosomes present in females while males are ZZ. Moreover, and quite surprisingly, recent evidence shows that birds do not have dosage compensation of Z chromosome genes [8,9].

The draft sequence of the chicken (*Gallus gallus*) genome [10] provided a starting point for evolutionary genomic analyses of birds. For example, it was found that the rate of synonymous substitution (*d*_*S*_) correlates negatively with chromosome size [11], something that may be related to GC content and recombination rate, which are both also negatively correlated with chromosome size. Moreover, the heterogeneous nature of the rate of recombination across avian chromosomes seems to have a significant effect on the evolution of base composition, reinforcing the heterogeneity in GC content (isochores) [12], which contrasts with the situation in mammals where isochores are generally decaying [13]. More recently, there have been initial attempts toward identifying genes subject to positive selection in avian lineages [14] and quantification of adaptive evolution in avian genes and genomes [15].

Now the genome of a second avian species, the zebra finch (*Taeniopygia guttata*), has been sequenced and assembled [16]. With this additional reference point, comparative genomic analysis of evolutionary processes in birds can begin in earnest. In this study we analyzed the molecular evolution of all known single-copy protein-coding genes shared by the chicken, zebra finch and mammalian genomes. We compared rates of sequence divergence and protein evolution in chicken and zebra finch lineages as well as in the ancestral bird branch leading from the split between birds and lizards some 285 million years ago. We looked for signals of selection to identify interesting genes for functional studies, similar to previous scans for positively selected genes in the human genome [17,18].

Additionally, we paid special attention to zebra finch orthologs of genes that have known significance in human learning, neurogenesis and neurodegeneration, using information in the Online Mendelian Inheritance in Man (OMIM) database. The zebra finch is an important model organism for these aspects of neuroscience [19,20]), and indeed this was a major motivation for the decision to determine its genome sequence [21]. The zebra finch is a songbird, one of several thousand oscines in the order Passeriformes. Songbirds communicate via learned vocalizations, under the control of a unique circuit of interconnected brain nuclei that evolved only in songbirds but have parallels in the human brain [22-24]. Studies of vocal learning in songbirds have revealed roles for lifelong neuronal turnover (neurodegeneration and neurogeneration) in the adult brain [19,20]. Hence, it is worthwhile to assess the evolutionary relationships of genes potentially involved in these processes in both humans and songbirds.

## Results

### Pairwise comparison of the chicken and zebra finch protein-coding gene sets

We identified 11,225 1:1 orthologs from the pairwise comparison of all protein-coding genes in the chicken and zebra finch draft genome sequences. This corresponds to 60 to 65% of the total number of genes in the avian genome [10]. The overall degree of neutral divergence, as approximated by the rate of synonymous substitution (*d*_*S*_) from 1,000 random sets of 150 genes [25], between these two bird species was 0.418 (95% confidence interval = 0.387 to 0.458). The overall ω (*d*_*N*_/*d*_*S*_) in the pairwise comparison was 0.152 (95% confidence interval = 0.127 to 0.179).

### Lineage-specific rates of evolution

For most of the subsequent analyses we used codon-based multiple species alignments of 8,384 1:1 orthologs of chicken, zebra finch, *Anolis *(lizard), and three mammals, including platypus, opossum, human or mouse (see phylogeny in Figure S1 in Additional file 1), thereby allowing lineage-specific estimates of rates of evolution. The rationale for focusing on single-copy genes was that we sought to avoid problems arising from the establishment of orthology/paralogy within gene families of birds and/or mammals. The estimates are sensitive to procedures for alignment and the substitution rate models used; see Additional file 2 for a justification of the methods applied here. Table 1 summarizes the estimates of mean *d*_*N*_, *d*_*S *_and ω using a free-ratio model for: (i), the ancestral bird lineage from the split between birds and lizards some 285 million years ago (MYA) [26] until the split between the chicken (Galloanserae) and zebra finch (Neoaves) lineages, for which we use an estimate of 90 MYA [27]; (ii), the chicken lineage; and (iii), the zebra finch lineage since the split between Galloanserae and Neoaves (Figure S1 in Additional file 1).

**Table 1:** Summary statistics of the overall rate of non-synonymous (*d*_*N*_) and synonymous (*d*_*S*_) substitution, and their ratio (ω) in avian lineages

	Pairwise chicken-zebra finch	Zebra finch	Chicken	Ancestral bird lineage
Overall *d*_*N*_	0.0635	0.0283	0.0239	0.0288
	(0.0517-0.0777)	(0.0225-0.0350)	(0.0185-0.0316)	(0.0241-0.0345)
Overall *d*_*S*_	0.4184	0.2133	0.1973	0.2600
	(0.3868-0.4584)	(0.1929-0.2384)	(0.1810-0.2154)	(0.2361-0.2834)
Overall ω	0.1517	0.1326	0.1208	0.1107
	(0.1270-0.1788)	(0.1080-0.1601)	(0.0973-0.1527)	(0.0942-0.1295)

95% confidence intervals based on resampling are given in parentheses.

*d*_*S *_was significantly (8%) higher in the zebra finch (0.213) than in the chicken lineage (0.197; *P *< 2.2 × 10^-16^, Wilcoxon signed rank test; Table 1), indicating a difference in the molecular clock of these two parallel lineages. *d*_*S *_of the ancestral bird lineage was higher (0.260) than in the two terminal branches, which is not unexpected given the estimated divergence times. The divergence at fourfold degenerate sites showed the same trend, and was highest in the ancestral bird lineage (mean of 1 Mb intervals = 0.239), and higher in zebra finch (0.199) than in chicken (0.172). We estimated lineage-specific mutation rates by dividing the divergence at fourfold degenerate sites with the estimated age of lineages according to the divergence times given above. We found that the mutation rate was lower in the ancestral bird lineage (1.23 × 10^-9 ^site^-1 ^year^-1^)than in both the chicken lineage (1.91 × 10^-9 ^site^-1 ^year^-1^; *P *< 2 × 10^-16^) and the zebra finch lineage (2.21 × 10^-9 ^site^-1 ^year^-1^; *P *< 2 × 10^-16^), and that the rate in the chicken lineage was significantly lower than the rate in the zebra finch lineage (*P *< 1 × 10^-5^).

The divergence at fourfold degenerate sites of orthologous genes was significantly correlated between zebra finch and chicken on the basis of 1 Mb windows, explaining 13 to 14% of the among-windows variance (Table 2). The correlations involving the ancestral lineage were weak and non-significant. Since local GC content is also conserved between zebra finch and chicken, controlling for GC content (see Materials and methods) strongly reduced the correlation between zebra finch and chicken divergence (from *r^2 ^*= 0.134 and 0.141 to *r^2 ^*= 0.024 and 0.019 for the zebra finch and chicken, respectively; Table 2).

**Table 2:** Correlations of divergence at fourfold degenerate sites between avian lineages in 1-Mb windows

	Without controlling for GC				Controlling for GC		
		
	*R*	d.f.	*P*	*r* ^2^	*r*	*P*	*r* ^2^
Windows based on zebra finch genome							
Zebra finch/chicken	0.366	441	1.89 × 10^-15^	0.134	0.156	0.001	0.024
Zebra finch/ancestral	-0.048	441	0.309	0.002	-0.146	0.002	0.021
Chicken/ancestral	0.074	441	0.119	0.005	-0.046	0.331	0.002
							
Windows based on the chicken genome							
Chicken/zebra finch	0.778	438	3.71 × 10^-16^	0.141	0.138	0.004	0.019
Chicken/ancestral	0.073	438	0.017	0.013	-0.008	0.868	0.000
Zebra finch/ancestral	-0.064	438	0.180	0.004	-0.161	0.001	0.026

d.f., degrees of freedom.

The zebra finch lineage had a significantly higher overall ω than the chicken lineage (0.133 versus 0.121; *P *< 2.2 × 10^-16^, Wilcoxon signed rank test). Just as for divergence, there was a strong correlation between individual ω values of 1:1 chicken and zebra finch orthologs (*r*^2 ^= 0.338, *P *< 2 × 10^-16^). A corresponding analysis for 7,789 human and mouse orthologs (included in the 8,384 genes from multiple-species alignments) revealed a similarly strong correlation (*r*^2 ^= 0.359, *P *< 2 × 10^-16^). Moreover, we also found a similar strength of correlation in gene-wise ω values estimated for orthologs from the bird lineage (chicken and zebra finch) with the mammalian lineage (human and mouse lineages; *r*^2 ^= 0.325, *P *< 2 × 10^-16^). The gene-wise correlations between ω values for the ancestral bird lineage (which had an overall ω of 0.110) and chicken (*r*^2 ^= 0.178, *P *< 2 × 10^-16^) and zebra finch (*r*^2 ^= 0.170, *P *< 2 × 10^-16^), respectively, were weaker.

### Adaptive evolution of genes in the avian genome

We next sought to identify genes, and the functional categories these genes are associated with, that are candidates for being involved with lineage-specific adaptations during avian evolution. We considered the ancestral bird lineage as well as the terminal chicken and zebra finch lineages separately, and posed three specific questions.

First, which genes have evolved most rapidly in avian lineages (high ω values), indicative of either adaptive evolution or relaxed selective constraint? For this question we used a likelihood ratio test to determine which genes had a significantly higher ω value than the mean of all genes in the genome. These genes are referred to as rapidly evolving bird (REB) genes. We used this approach rather than simply selecting, for example, the top 5% or 10% of genes sorted by ω value since the confidence in ω values is dependent of alignment length and the number of substitutions within a particular gene.

Second, which genes have evolved more rapidly in avian lineages than in other amniote lineages (mammals and lizard)? Here we used a branch model in PAML to determine which genes had a significantly higher ω in avian lineages than in other branches of the tree corresponding to our data. These genes are referred to as more rapidly evolving in birds (MREB).

Third, which genes show evidence of containing codons that have been subject to positive selection (referred to as PS genes) during avian evolution? For this third question we used a branch-site model in PAML to identify genes containing positively selected codons with ω higher than 1.

In total, 1,751 genes were identified as evolving significantly more rapidly than the genomic average (REB) in one or more of the three avian lineages (Table 3). Of these REB genes, 203 (12%) were common to all three lineages (Figure S2 in Additional file 1); 1,649 genes showed evidence of more rapid evolution in one or more bird lineages (MREB) than in other amniotes (Table 3). The great majority (>97%) of these genes were specific to a single bird lineage, with no gene common to all three lineages (Figure S2 in Additional file 1). We also identified 1,886 PS genes in avian lineages (Table 3). Most (>85%) of these genes showed evidence of positive selection in only a single lineage (Figure S2 in Additional file 1). As for the REB category, it may contain genes that evolve rapidly due to positive selection but also due to relaxed constraint. Using randomization tests, we compared the number of overlapping genes between the REB and PS gene lists with the number of overlapping genes from gene lists generated randomly. For all three avian branches (zebra finch, chicken, and ancestral bird lineages), the number of overlapping genes between the PS and REB gene lists is significantly higher than in randomized data sets (*P *< 0.001 for all three branches). This shows that the genes that we identified as rapidly evolving are unlikely to be dominated by genes evolving under relaxed constraint.

**Table 3:** The number of REB, MREB and PS genes in different avian lineages

	Ancestral lineage	Chicken lineage	Zebra finch lineage
Rapidly evolving bird (REB) genes	419	1,148	1,202
More rapidly evolving genes in birds (MREB) than in other amniotes	103	432	1,154
Positively selected (PS) bird genes	259	883	936

The lists of REB, MREB and PS genes will constitute a useful resource for future research aimed at finding the genetic basis of adaptive evolution in birds, in particular the list of PS genes. Here we provide an initial characterization of genes from these lists by first testing for an over-representation of specific gene ontologies (Table 4). The term 'cell adhesion' was over-represented among REB, MREB as well as PS genes in the ancestral bird lineage. Terms related to ion-channel activity were over-represented among PS genes in both the ancestral bird and chicken lineages. The ancestral lineage also showed an over-representation of the terms blood vessel development, synapse organization, integrin-mediated signaling pathway and proteinaceous extracellular matrix among MREB genes and of cytokine secretion among REB genes. In the chicken lineage, telomere organization and sterol transport were enriched among REB genes while in the zebra finch lineage microtubule cytoskeleton was over-represented among MREB genes. Table S1 in Additional file 1 lists all genes corresponding to significantly over-represented Gene Ontology (GO) terms.

**Table 4:** Over-represented Gene Ontology terms in REB, MREB and PS genes in avian lineages

	Ancestral bird lineage				Chicken lineage				Zebra finch lineage			
			
Gene Ontology^a^	N_1_^b^	N_2_^c^	Excess	*P*	N_1_^b^	N_2_^c^	Excess	*P*	N_1_^b^	N_2_^c^	Excess	*P*
Rapidly evolving in birds (REB)												
Biological adhesion (B 2)	17	136	2.67	0.013								
Cell adhesion (B 3)	17	135	2.69	0.013								
Cytokine secretion (B 7)	4	5	17.06	0.013								
Telomere organization (B 5)					5	5	7.81	0.024				
Telomere maintenance (B 6)					5	5	7.81	0.024				
Sterol transport (B 5)					6	7	6.69	0.024				
Cholesterol transport (B 6)					6	7	6.69	0.024				
												
More rapidly evolving in birds (MREB) than in other amniotes												
Biological adhesion (B 2)	12	136	5.82	0.0002								
Cell adhesion (B 3)	12	135	5.86	0.0002								
Blood vessel development/maturation (B5)	2	2	65.94	0.061								
Synapse organization and biogenesis (B 5)	3	12	16.49	0.088								
Integrin-mediated signaling pathway (B 6)	3	11	17.98	0.088								
Proteinaceous extracellular matrix (C 3)												
Cytoskeletal part (C 5)									37	124	1.92	0.040
Microtubule cytoskeleton (C 7)									27	83	2.09	0.040
												
Positively selected (PS) in birds												
Biological adhesion (B 2)	16	148	3.27	0.016								
Cell adhesion (B 3)	16	147	3.29	0.016								
Cell-cell adhesion (B 4)	9	57	4.78	0.035								
Homophilic cell adhesion (B 5)	5	16	9.45	0.035								
Calcium ion binding (M 5)	14	154	2.74	0.035								
Anion transmembrane transport activity (M 6)					16	48	3.00	0.006				

Terms with a false discovery rate (FDR) of adjusted *P *< 0.1 are shown. Excess is the fold enrichment for significant Gene Ontology terms. ^a^B is biological process, M is molecular function and C is cellular component. The numbers indicate hierarchical level. ^b^Number of genes in test sample (REB, MREB and PS, respectively). ^c^Number of genes in reference sample (1:1 orthologs found in the respective lineage).

If positively selected codons are evenly distributed across genes and the power to detect such codons is more or less constant, then the likelihood of detecting genes containing positively selected codons will correlate with alignment length. Consistent with this, three out of three unique overrepresented GO terms from the list of positively selected genes in the ancestral bird branch have longer mean alignment length than genes with other GO terms (*P *< 0.001, Wilcoxon rank sum test). However, the overrepresented GO terms from the list of positively selected genes in the chicken lineage have actually shorter mean alignment length than genes with other GO terms, with marginal significance (*P *= 0.093). This warrants further investigation, from both methodological and biological points of view.

As a comparison, we tested for over-represented GO terms among positively selected mammalian genes and genes evolving significantly faster in mammals than in birds (Table S2 in Additional file 1). However, using the same criteria as applied to the lists of avian genes, no GO term was significantly over-represented in the mammalian lists.

### Adaptive evolution of neurological genes

The lineage leading to the zebra finch and other passerine birds is distinguished from the chicken lineage by major neurobehavioral adaptations that have parallels in humans, including the evolution of vocal communication as well as other forms of learning, memory and social cognition [28]. We filtered the lists of positively selected genes in the zebra finch and chicken lineages to identify candidate genes likely to contribute to evolution of these traits. We began by considering the orthologs of genes that have been most strongly implicated in learning and neuronal plasticity in humans, identifying them by searching the OMIM database for all genes associated with 'learning', 'neurogeneration' or 'neurodegeneration'. We had data from multispecies alignments for 74, 211 and 107 such genes, respectively (Table 5). We found that 15, 34 and 23 of these genes (in total, 58 unique genes) were present in the list of 1,036 genes identified as positively selected in the zebra finch lineage (Table 5; Table S3 in Additional file 1). For the term 'neurodegeneration' in particular, the number of positively selected genes is significantly higher than expected by chance (*P *= 0.0076, Fisher's exact test) given the overall frequency of positively selected genes among all genes in our study.

**Table 5:** OMIM search for genes implicated in neurological processes and the number of these identified as evolving under positive selection in the chicken and zebra finch lineages

Search term*	N_OMIM_	N_align_	PS_chicken_	PS_zebra_	*P*
Learning	159	74	5	10	0.050
Neurogenesis	472	211	15	27	0.017
Neurodegeneration	246	107	8	16	0.025

*See Materials and methods. 'N_OMIM_' is the number of human genes identified in OMIM, 'N_align_' is the number N_OMIM _genes for which we had data from multispecies alignments. 'PS_chicken_' and 'PS_zebra_' are the number of unique positively selected genes found in the chicken and zebra finch lineages, respectively. *P *is the significance level in Fisher's exact test comparing the incidence of positively selected genes in chicken and zebra finch.

We then compared the number of genes classified as associated with 'learning', 'neurogeneration' or 'neurodegeneration' that were found to be positively selected in either the chicken or zebra finch lineage (that is, excluding genes that were positively selected in both lineages). Interestingly, for each OMIM term the number of unique positively selected genes was significantly higher in zebra finch than in chicken (Table 5; 10 versus 5, 27 versus 15, and 16 versus 8, respectively). This indicates that the songbird lineage has experienced more frequent adaptive evolution of genes relating to cognitive functions than the galliform lineage.

The 58 neurological genes evolving under positive selection in the songbird lineage were further assessed in two ways. First, we asked whether any of them also show evidence of accelerated sequence evolution in the primate lineage, using data from the study of Dorus *et al*. [29]. Four genes are present on both lists: *ASPM*, *GRIN2a*, *DRD2*, and *LHX2 *(Table 6). Second, we asked whether any of them are also expressed differentially within the songbird-specific song control nuclei of the zebra finch brain. Lovell *et al*. [30] used a combination of microarray and *in situ *hybridization analyses to identify approximately 300 genes that are differentially expressed in the song nucleus high vocal centre (HVC) compared to the underlying brain tissue. We found that 9 of our 58 neurological genes evolving under positive selection are also differentially regulated in the high vocal centre (Table 6), including glutamate receptor ion channel genes.

**Table 6:** Genes implicated in neurobehavioral evolution by converging lines of evidence

Ensembl ID	Locus	Gene
Evolving rapidly in the primate lineage [29]		
ENSTGUG00000000255	*DRD2*	D(2) dopamine receptor
ENSTGUG00000004249	*ASPM*	Abnormal spindle-like microcephaly-associated protein
ENSTGUG00000004747	*GRIN2A*	Glutamate [NMDA] receptor subunit epsilon-1 precursor
ENSTGUG00000007079	*LHX2*	LIM/homeobox protein Lhx2
		
Differentially expressed in zebra finch song control system [30]		
ENSTGUG00000000694	*GPR98*	G protein-coupled receptor 98 precursor
ENSTGUG00000002176	*MCF2*	Mcf2 transforming sequence-like
ENSTGUG00000004464	*NEFL*	Neurofilament triplet L protein
ENSTGUG00000005484	*GRIA2*	Glutamate receptor, ionotropic AMPA 2
ENSTGUG00000006839	*CACNA1D*	Voltage-dependent L-type calcium channel subunit alpha-1D
ENSTGUG00000007224	*PTPRF*	Protein tyrosine phosphatase receptor type F
ENSTGUG00000007343	*RAI1*	Retinoic acid-induced protein 1
ENSTGUG00000010757	*GRM1*	Glutamate receptor, metabotropic 1
ENSTGUG00000015209	*SYCP1*	Synaptonemal complex protein 1

Neurological genes under positive selection in the zebra finch (see also Table S3 in Additional file 1) were assessed for representation in the results of two other studies: orthologs under positive selection in the primate lineage (Dorus *et al*. [29]) and zebra finch genes that are differentially expressed in song nucleus the high vocal centre compared to the underlying 'shelf' region (Lovell *et al*. [30]).

### The relationship between selection and recombination

We sought to elucidate how the intensity of selection and/or the influence of genetic drift, manifested in ω, vary across the avian genome. The potential influence of recombination on ω was of particular interest since the rate of recombination is unusually heterogeneous within both the chicken [31] and zebra finch [32] genomes, and probably so for birds in general. Such heterogeneity could set the stage for recombination affecting the efficacy of selection and thereby ω, as predicted by evolutionary theory [33] but for which there is limited empirical support [34-38].

**Figure 1:**
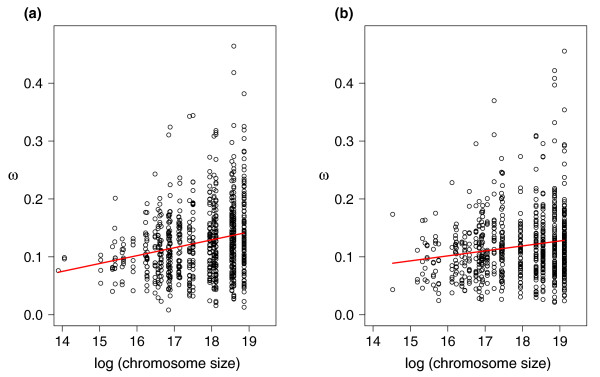
**The relationship between ω estimated for 1-Mb intervals and chromosome size**. **(a) **Zebra finch; **(b) **chicken.

**Figure 2:**
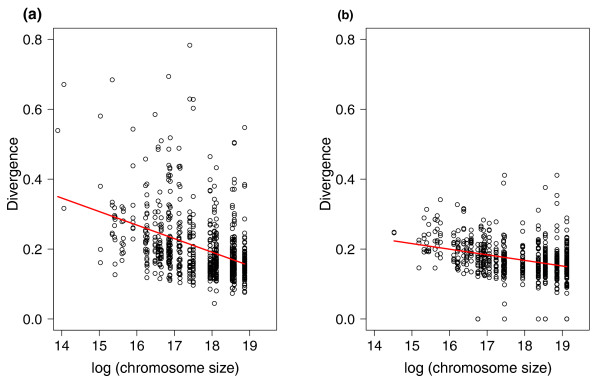
**The relationship between the mean mutation rate (divergence at fourfold degenerate sites) for 1-Mb intervals and chromosome size**. **(a) **Zebra finch; **(b) **chicken.

As a starting point for these analyses we first noted that there was a weak positive correlation between ω estimated for 1 Mb intervals and chromosome size in zebra finch (Figure 1; *r*^2 ^= 0.055, *P *= 6 × 10^-11^) and chicken (*r*^2 ^= 0.029, *P *= 3 × 10^-6^). This confirms similar observations made for a small set of chicken-turkey orthologs [11] as well as for chicken-human orthologs [10], although the effect we detected here with much larger data sets was considerably weaker than indicated by those previous studies. There was a strong negative correlation between the mean divergence of fourfold degenerate sites of 1 Mb intervals and chromosome size (Figure 2; *r*^2 ^= 0.153 in zebra finch and *r*^2 ^= 0.140 in chicken, *P *< 2 × 10^-16 ^in both cases). These correlations were not limited to the dichotomy of macrochromosomes versus microchromosomes (data not shown); indeed, for many birds chromosome size shows a relatively continuous distribution without a clear distinction between macrochromosomes and microchromosomes [7].

We found a weak yet statistically significant negative relationship between recombination rate and ω in both zebra finch (Table 7; *r*^2 ^= 0.030, *P *= 4 × 10^-5^) and chicken (*r*^2 ^= 0.011, *P *= 0.005). This could possibly be related to other factors co-varying with these parameters. For example, GC is strongly correlated with recombination rate in both chicken [31] and zebra finch [32], and in our data GC content correlates negatively and weakly with ω (zebra finch, *r*^2 ^= 0.017, *P *= 0.002; chicken, *r*^2 ^= 0.005, *P *= 0.068). GC content might be correlated with ω because biased gene conversion tends to increase ω due to an increased rate of fixation of slightly deleterious alleles, mimicking adaptive evolution [39], and higher GC content tends to decrease the number of synonymous sites [40,41]. Moreover, gene density is higher in avian microchromosomes than in macrochromosomes [10] and there are strong correlations between chromosome size and both GC and recombination rate [31]. Gene density might be critical to the effects of recombination on the efficacy of selection because more coding sequence should, in principle, imply more targets for selection. When we tested for a correlation between recombination rate and ω at the same time as controlling for GC and gene density (proportion of coding sequence within 1 Mb windows), we still found weak yet significant negative relationships (chicken, *r*^2 ^= 0.006, *P *= 0.032; zebra finch, *r*^2 ^= 0.008, *P *= 0.031). The effect is not limited to regions with very low recombination rate as similar results were obtained when comparing windows with zero and non-zero recombination rates (data not shown).

**Table 7:** Bivariate and partial correlations (with GC content and amount of coding sequence controlled for) between ω and recombination rate in 1 Mb windows

	*t*	d.f.	*P*	*r* ^2^
Zebra finch				
Bivariate	-4.13	557	0.00004	0.030
Controlled for GC	-2.8	556	0.0053	0.014
Controlled for CDS	-4.51	556	0.00001	0.035
Controlled for GC and CDS	-2.16	555	0.0313	0.008
				
Chicken				
Bivariate	-2.82	713	0.0049	0.011
Controlled for GC	-2.15	712	0.0320	0.006
Controlled for CDS	-2.44	712	0.0149	0.008
Controlled for GC and CDS	-2.14	711	0.0329	0.006

CDS, coding sequence; d.f., degrees of freedom; *t*, t-statistic (t-score) of the slope.

## Discussion

Modern birds form two monophyletic clades, the Palaeognathae (ratites, like ostrich and its allies) and the Neognathae (the great majority of contemporary bird species), which diverged during the cretaceous between 80 and 130 MYA [42-45]. Within the Neognathae, the first split was between Galloanserae (fowl-like birds (including chicken), ducks and geese) and Neoaves (>20 different orders) [46,47]. Diversification within Neoaves seems to have occurred rapidly, with very short internal nodes in the basal part of the Neoaves tree [45,48]. One of these early offshoots within Neoaves was the order Passeriformes, to which zebra finch belongs. These birds typically have small body size and are relatively short-lived compared to chicken and their allies within Galloanserae.

When judged from the divergence at fourfold degenerate sites across more than 8,000 genes, the mean mutation rate in birds was 1.23 to 2.21 × 10^-9 ^site^-1 ^year^-1^. The rate was lowest in the ancestral bird lineage from the split between birds and lizards until the split between Galloanserae and Neoaves (1.23 × 10^-9 ^site^-1 ^year^-1^), was intermediate in the chicken lineage (1.91 × 10^-9 ^site^-1 ^year^-1^) and was highest in the zebra finch lineage (2.21 × 10^-9 ^site^-1 ^year^-1^). This indicates a rate acceleration among modern birds and particularly so in Neoaves, or more specifically, in the lineage leading to zebra finch. The difference in mutation rate between the chicken and zebra finch lineages is in a direction predicted by a generation time effect [49]: shorter generation times among small songbirds may have led to higher per-year mutation rates. We note that this inference relies on the underlying assumption of neutrality of fourfold degenerate sites. To the best of our knowledge there is no evidence for codon usage bias in avian genes; if it exists, it seems unlikely that selection for codon usage on a genome-wide scale would differ among the investigated lineages to an extent that can explain the almost twofold higher mutation rate in the zebra finch compared to the ancestral lineage.

The lower mutation rate estimated for the ancestral bird branch is sensitive to the accuracy of the estimated divergence times of birds and lizards (285 MYA), and of Galloanserae and Neoaves (90 MYA). Previous molecular datings of the Galloanserae-Neoaves split have provided estimates in the range of 90 to 126 MYA, with a mean of 105 MYA [50]. Using this mean value, instead of 90 MYA, to estimate the substitution rate still leads to a faster rate in modern birds than in the ancestral bird branch (zebra finch, 1.90 × 10^-9 ^site^-1 ^year^-1^; chicken, 1.63 × 10^-9 ^site^-1 ^year^-1^; ancestral birds, 1.33 × 10^-9 ^site^-1 ^year^-1^). The earliest divergence estimate of 126 MYA leads to similar substitution rates in the ancestral and zebra finch lineages. However, such an old divergence is not supported by the fossil record, which indicates a split younger than 100 MYA [42,44]. Importantly, not a single modern bird is known in the lower cretaceous (145 to 100 MY) despite a reasonably good fossil record [43,51,52]. Another potential concern is that, because of saturation (that is, when multiple substitutions impair the model to reliably estimate substitution rates), the ancestral branch length may have been underestimated. It is difficult to directly assess the possible effect of saturation on the length of the ancestral bird branch. However, we note that a similar trend (lower rate of divergence in the ancestral branch) is not evident among eutherian mammals from the same set of genes (Table S4 in Additional file 1).

The ancestral lineage from the split between birds and lizards until the split between Galloanserae and Neoaves represents, for the most part, dinosaurs that existed before the appearance of modern birds (*Archaeopteryx *fossils date back around 145 MYA). If the estimated mutation rates are correct and if one assumes a generation time effect, our data would suggest that generation times in the saurischian dinosaur lineage were typically longer than in modern birds.

Previous studies of divergence in mammalian genomes have indicated a low degree of substitution rate conservation over evolutionary time scales comparable to that between chicken and zebra finch, for example, in the comparison between primate and rodent lineages [53,54]. These estimate have been based on interspersed repeat elements under the (reasonable) assumption that these sequences are selectively neutral. Our analysis of divergence at fourfold degenerate sites between orthologous regions of chicken and zebra finch revealed a stronger correlation, with 13 to 14% of the variation in divergence in one lineage explained by variation in divergence in the other. This could reflect that the selective constraints on fourfold degenerate sites and interspersed elements differ (being higher in fourfold degenerate sites) so that the two approaches are not directly comparable. Alternatively, there might be biological explanations for high mutation rate conservation in birds. When controlling for the local GC content, the amount of variation in divergence explained by the orthologous rate is reduced to 2%. This shows that avian mutation rate conservation is largely dependent of conservation in base composition. Compared to mammalian genomes, avian GC content is highly heterogeneous and this heterogeneity has been maintained during avian evolution [12]. It was suggested that the heterogeneous recombinational landscape of birds [12] reinforces GC heterogeneity via biased gene conversion. Local recombination rates are significantly correlated between chicken and zebra finch [32] and it may very well be that there is a causal connection between conservation in recombination, base composition and mutation rate [55-57].

### Over-represented gene ontologies among positively selected or rapidly evolving genes

With draft sequences now available for two avian genomes it is possible to study the role of natural selection in shaping individual gene sequences during avian evolution. An impetus for our study was thus to identify genes and gene categories that have been important for adaptive character evolution in a vertebrate lineage. Clearly, there are many morphological, physiological and behavioral phenotypes that distinguish birds and mammals. A comparative genomic approach has the potential to contribute towards the identification of the genetic basis of these differences [58].

Basic characteristics of birds such as feathers, flight and hollow bones evolved prior to the split of the chicken and zebra finch lineages. The genetic novelties underlying these phenotypes should thus have started to appear in an ancestral lineage. As discussed above, the ancestral bird branch in the phylogenetic tree formed by our data corresponds mostly to non-avian dinosaurs of the order Saurischia, suborder Theropoda. Genes or gene categories identified as positively selected or rapidly evolving in this branch may thus be related to phenotypic evolution in non-avian dinosaurs rather than in modern birds. On the other hand, many bird-like features may have started to emerge already for non-avian dinosaurs.

The two GO terms found to be over-represented among genes evolving under positive selection in the ancestral bird lineage, calcium ion binding and cell adhesion, largely represent an overlapping set of genes. Most of these genes (Table S1 in Additional file 1) encode transmembrane cadherins that play a critical role in cell-cell adhesion in tissue structures. One of these cadherins, protocadherin-15, is expressed in retina and we note that another positively selected calcium ion binding gene, Crumbs homolog 1, is involved with photoreceptor morphogenesis in retina; mutations in the human ortholog cause retinitis pigmentosa type 12 [59]. The visual ability of birds is superior to other vertebrates and the molecular adaptations underlying this phenotype are likely to have been driven by positive selection.

In the chicken lineage the term anion transmembrane transporter activity was over-represented among positively selected genes. The genes annotated with this term include solute carriers and ion channels involved with basic cell signaling processes, for example, in neurotransmission. In the zebra finch lineage the term microtubule cytoskeleton was over-represented among genes evolving faster in this lineage than in other branches of the amniote tree. The majority of these are kinesins and other genes involved with mitosis/meiosis, sperm motility, centrosome formation and synapse function.

It should be stressed that we inferred positive selection in lineages corresponding to nearly 100 million years or more of evolution and that large numbers of genes were uncovered by these analyses. This is likely to reduce the power of detecting enriched GO terms due to dilution and failure to capture temporal episodes of adaptive evolution. Moreover, given that our data were defined by a common set of 1:1 orthologous genes found in birds, a lizard and mammals, the analysis did not include lineage-specific genes that may be particularly responsive to positive selection. These aspects are probably of relevance to the somewhat surprising observation that no significantly over-represented GO terms were found among positively selected or rapidly evolving mammalian genes. This is seemingly at odds with previous work in primates that frequently have revealed categories such as sensory perception, immune defence, apoptosis and spermatogenesis to be enriched among positively selected genes [17,18,60-62]. In birds, there have recently been large-scale efforts toward transcriptome sequencing of several species, including songbirds [63]. These data will allow study of the molecular evolution of genes in much shorter branches of the avian phylogenetic tree than is currently possible with complete genome sequences, which is only available for chicken and zebra finch.

### Zebra finch and positive selection in neurological genes

The zebra finch communicates through learned vocalizations ('songs'). Only the male zebra finch produces learned song, and he learns this song by copying an adult tutor during a critical period in juvenile development. Experimental work in zebra finch has demonstrated the localization and character of neural circuits involved in developmental song learning and adult singing [64-67], with dynamic regulation of brain gene expression in response to singing and song experience [68-76]. Fifty-eight genes with known roles in learning, neurogenesis or neurodegeneration in humans show evidence of positive selection in the zebra finch lineage. Of these, nine (15%; Table 6) are also expressed differentially in the song control system, either at higher or lower levels than in the surrounding brain tissue, according to the study of Lovell *et al*. [30]. In comparison, only 2% (390 out of 17,214) unique brain-derived cDNA probes on that microarray gave differential hybridization signals in the song control system. We note that five of the nine genes encode proteins involved in cell surface and synaptic signaling: voltage-dependent L-type calcium channel subunit alpha-1D (*CACNA1D*), G protein-coupled receptor 98 precursor (*GPR98*), glutamate receptor, ionotropic AMPA 2 (*GRIA2*), glutamate receptor, metabotropic 1 (*GRM1*), and protein tyrosine phosphatase receptor type F (*PTPRF*). *GRIA12 *is also one of the ion channel genes that are suppressed in response to song playbacks as reported in Warren *et al*. [16].

Four of the 58 genes show evidence of accelerated evolution in the primate lineage: *ASPM*, *GRIN2a*, *DRD2*, and *LHX2*. Two of these have apparent roles in neurogenesis and neuronal development (*ASPM *and *LHX2*). In particular, *ASPM *(abnormal spindle-like microcephaly-associated) has been a focus of speculation with respect to the dramatic evolution of brain size in humans. Homozygous mutations in *ASPM *are a cause of primary microcephaly [77] and the gene shows evidence of positive selection in both the human lineage [78-80] and the ancestral lineage of the apes [81]. Songbirds have also experienced a relative increase in brain size compared to other avian lineages [82], with the notable emergence of the large and highly plastic nuclei of the song control system. However, enthusiasm for *ASPM *as a key factor in primate brain evolution has been tempered by findings that mutations in *ASPM *are not correlated with cognitive ability [83,84] and by alternative roles for *ASPM *that might place it under selection more broadly, such as a role in ciliary function [85].

The other two neurological genes that are also accelerated in primates may be considered to have neuromodulatory functions that can directly affect learning, memory and behavior. *DRD2 *encodes the D2 subtype of the dopamine receptor. *GRIN2a *encodes a subunit of the N-methyl-D-aspartate (NMDA) receptor, a subtype of ionotropic glutamate-gated ion channel that has well-established roles in learning and brain plasticity (reviewed in [86]). A survey of *GRIN2a *sequences across primate species revealed a specific correlation between ω and home range size, which is taken to be a proxy for spatial memory [87]. Spatial memory is well developed in the songbird (passerine) lineage and is especially evident in food-caching species [88], a behavior that depends on NMDA receptor function [89]. Zebra finches are not studied as a food caching species, but their nomadic lifestyle implies a highly sophisticated spatial sense [90]. NMDA receptors have also been implicated in song learning and song control system neurophysiology [91,92]. The rich diversity of songbird species and their adaptations should provide unusual opportunities for correlating NMDA receptor sequence evolution with specific behavioral and neurophysiological variations.

### The strength of selection during avian evolution

The overall strength of selection as manifested in the genome-wide ratio of non-synonymous to synonymous substitution rates (ω) was similar in the chicken and zebra finch lineages (0.12 to 0.13), as well as in the ancestral bird lineage (0.11). These ratios are about half that reported among hominids and more similar to what is seen in the murid and dog lineages [62]. This may be taken to suggest that the rate of adaptive evolution and/or the rate of accumulation of slightly deleterious mutations have been lower in birds than in primates. However, it is increasingly appreciated that point estimates of mean ω can be misleading. Mean ω decreases with branch length and needs to be seen in a time trajectory framework rather than as a fixed quantity [93-95]. The apparent lineage-specific differences between hominids on the one side and murids, dogs and birds on the other may thus simply be accounted for by branch length. Future research will be needed to explore how branch length is best accounted for when comparing mean ω for different lineages. This will be important when addressing whether life history variables, such as the effective population size (N_e_), correlate with mean ω. For example, such a correlation might be expected if slightly deleterious mutations contribute significantly to protein evolution as postulated by a nearly neutral model [96], giving rise to a negative relationship between mean ω and N_e _[97].

### The relationship between natural selection and recombination in avian genomes

Selection acts in each generation on alleles embedded within a particular genomic background. Due to recombination, selection will, over time, be able to favor or disfavor alleles at individual loci without affecting the rest of the genome. This comes with a caveat that when two loci are genetically linked, selection at one locus will affect the efficiency of selection at the other: the loci are said to interfere with each other. Theory predicts that the strength of interference should be related to the amount of recombination between the loci; this is the so-called Hill-Robertson effect [33]. Theoretical predictions on the consequence of Hill-Robertson interference on coding sequence evolution depend on the fitness distribution of segregating variants at non-synonymous sites [98,99]. If slightly deleterious mutations segregate frequently in the population, directional selection at one locus will increase the probability of fixation of such mutations at linked loci. If beneficial alleles are common in the population, the probability of fixation of those mutations will be reduced at linked loci. These two scenarios are associated with opposing predictions for the correlation between recombination rate and ω; in the former case a negative relationship is expected whereas in the latter case a positive relationship is expected.

The strongest support for Hill-Robertson interference comes from regions devoid of recombination. For example, ω is generally high in the non-recombining sex chromosome, that is, the Y chromosome in systems with male heterogamety and the W chromosome in systems with female heterogamety [100-102]. However, it has been surprisingly difficult to find genome-wide empirical support for Hill-Robertson interference, and data are currently limited to studies in *Drosophila *[34,35,103,104] and a recent study of humans failed to demonstrate a correlation between recombination rate and ω [37].

It is possible that the power for detecting a relationship between recombination and ω could be higher in bird systems because the rate of recombination is highly heterogeneous, at least within the two avian genomes for which detailed information is currently available on regional recombination rate variation. Specifically, there is a clear negative relationship between chromosome size and recombination rate [10,31] following from an obligate recombination event per chromosomal arm. In chicken, the average per-chromosome recombination rate ranges from 2 centiMorgans (cM)/Mb up to 10 cM/Mb [10]. Moreover, there is significant within-chromosome variation in the rate of recombination with a strong 'telomere effect'. This is most readily seen in zebra finch, with rates close to 10 cM/Mb in terminal regions of the larger (>100 Mb) chromosomes while the central parts have rates as low as 0.1 cM/Mb; the latter is not just a 'centromere effect' because these recombination deserts cover up to 75% of the larger chromosomes [32].

We do not find support for an increased efficiency of directional selection in regions of high recombination. If anything, the data go in the opposite direction since there was a weak negative, yet significant relationship between ω and recombination rate in both chicken and zebra finch (*r*^2 ^< 0.01, after controlling for GC content and the amount of coding sequence); this is the direction predicted from the hypothesis of an accumulation of slightly deleterious mutations in regions with low recombination rate. One obvious explanation for this weak relationship is that both slightly deleterious and beneficial variants are common and that their opposing effects in Hill-Robertson interference largely cancel each other out. However, in the absence of simulations under different distributions of the fitness consequences of segregation mutations this remains an argument that is difficult to examine.

Another explanation relates to the fact that recombination rate and ω are measured on very different time scales. Recombination is recorded from pedigree data and thus reflects the rate in contemporary populations. Lineage-specific ω represents substitutions that have accumulated during, in this case, 90 million years of avian evolution. If the recombination landscape has changed frequently during the course of this time period, this may have weakened the signal of potential recombination effects on the pattern of efficacy of selection across the genome. There is limited knowledge on the evolutionary consistency of regional recombination rate variation [105]. At a local scale, recombination hot-spots are ephemeral in the human genome with little or no evidence for hot-spots at orthologous positions in the chimpanzee genome [106-108]. As indicated above, recombination rates in birds are strongly associated with chromosome features, with highly elevated rates in microchromosomes and in telomeric regions of larger chromosomes. Given the high degree of karyotype stability in birds [7], this may suggest that the recombination landscape has also remained relatively stable. Indeed, we have found that recombination rates in 1-Mb windows of the chicken and zebra finch genomes to be significantly correlated [32]. Moreover, the strong correlation observed between base composition (GC content) and current recombination rates in both chicken [31] and zebra finch [32] is consistent with a conserved pattern of recombination rate variation, at least under the scenario that recombination drives the long-term evolution of base composition (maintenance of regions elevated in GC content) by biased gene conversion [57]. An alternative possibility is that base composition drives recombination rate variation and it is conservation of GC content, or GC-rich motifs [109], that results in the appearance of recombination rate conservation.

Further, the influence of Hill-Robertson interference on the accumulation of mildly deleterious substitutions is not expected to decrease linearly with an increase of the recombination rate [37,110]. In this context, it is possible that the recombination rate is too high in most regions of the chicken and the zebra finch genome to lead to measurable variation in the efficiency of selection. This would somewhat contradict the observation of very low recombination rates in the sub-centromeric region of the larger zebra finch chromosomes [32]. However, these recombination deserts can have a high effective number of recombination events given a very large population size, as is observed for natural zebra finch populations [111]. In general, it may very well be that the effective population sizes of ancestral passerines have been higher than that of other (larger) birds.

## Conclusions

We conducted a comparative analysis between two avian genomes using one lizard and three mammalian species as outgroups. Substitution rates were estimated from 8,384 1:1 orthologs of genes at fourfold degenerated sites and calibrated with the fossil record. We found clear substitution rate differences between the ancestral bird lineage and the lineage leading to chicken and to zebra finch, and argue that the differences possibly reflect an effect of generation time. We further report a list of positively selected and/or rapidly evolving genes in the abovementioned avian lineages. GO terms for several biological processes were over-represented among the positively selected genes, including anion transporter activity, calcium ion binding, cell adhesion and microtubule cytoskeleton. We highlight a set of 58 genes evolving under positive selection in the songbird lineage that are of particular interest in neurobiology. Nine of these genes are also differentially expressed in the unique vocal control nuclei of the songbird brain and may warrant special attention in the future. Finally, a significant but low negative relationship between recombination rate and ω supports the theoretical prediction that the efficiency of purifying selection may be reduced in regions of low recombination rate.

## Materials and methods

### Alignments

We downloaded protein-coding sequences from the chicken (*G. gallus*, WASHUC2), zebra finch (*T. guttata*, TaeGut3.2.4), green lizard (*Anolis carolinensis*, ANOCAR1), short-tailed opossum (*Monodelphis domestica*, MonDom5), platypus (*Ornithorhynchus anatinus*, OANA5), mouse (*Mus musculus*, NCBIM37) and human (*Homo sapiens*, NCBI36) genome assemblies through biomart [112] in Ensembl version 55. In order to identify 1:1 orthologs between zebra finch and each of the other species, we used a reciprocal Blast best hit approach as implemented in Inparanoid3.0 [113]. Codon-based pairwise alignments from the corresponding protein sequences were made using MUSCLE3.7 [114]. We used Gblocks 0.91b [115] to eliminate poorly aligned positions. In total, our analysis was based on 8,384 genes.

### Estimates of substitution rates

#### Pairwise rates

We used the codeml program in the PAML4.1 package [116] to estimate mean pairwise *d*_*S *_and ω (*d*_*N*_/*d*_*S*_) for all 11,225 1:1 orthologs of chicken and zebra finch from 1,000 concatenated alignments each constructed from 150 randomly chosen genes. Concatenation of alignments reduces the sampling variance by producing longer sequences for which parameters can be estimated more precisely [25]. The repeated sampling allows estimation of the within-genome variance (95% confidence intervals).

#### Fourfold degenerate rate

The neutral lineage-specific substitution rate in 1-Mb windows of the chicken and zebra finch genomes was approximated by estimating the divergence of fourfold degenerate sites (third codon positions of fourfold degenerated codons) using a GTR+ Gamma4 model of substitution with the baseml program in the PAML4.1 package. We based our analysis on windows with at least 1 kb of degenerate sites.

#### Lineage-specific substitution rates

We estimated lineage-specific mean *d*_*N*_, *d*_*S*_, and ω using the free-ratio model [117] in the same way as for the pairwise comparison, that is, applying the Heger and Ponting [25] method. Lineage-specific ω of individual genes was estimated using the branch model of PAML4.1, making the branch of interest foreground and collecting ω from this branch. This method has the advantage that it tends to show less sampling variance than a free-ratio model.

Mean ω values for 1-Mb windows were estimated by concatenating all alignments within each window and using the three-ratio model in codeml. This model was chosen to reduce the number of parameters and thus to avoid the problem of over-parameterization when small numbers of substitutions are analyzed. Windows were excluded if the alignment length was less than 1 kb or if the number of substitutions per window was fewer than 200. This approach avoids problems with decreased precision of estimates (higher sampling variances) when the number of substitutions is low.

The ω values for individual alignments were calculated using the three-ratio model in codeml. Alignments were excluded if *d*_*S *_> 2 or ω > 3 [14]. This analysis was based on 7,415 genes in birds and on 6,252 in eutherian mammals.

### Statistical models

We used bivariate and partial correlations to analyze the relationship between ω and recombination rate separately in chicken and zebra finch. The sex-average recombination rate for 1-Mb windows was obtained for chicken from Groenen *et al*. [31] and for zebra finch from Backström *et al*. [32]. Partial correlations controlled for GC content and the amount of coding sequence within each window individually and in combination. Similarly, we used bivariate and partial correlation (controlling for GC content) to study the association between divergence at fourfold degenerate sites from 1-Mb windows in different bird species. Since the windows were not identical between zebra finch and chicken, we estimated the correlations separately for zebra finch-chicken, and for chicken-zebra finch. The similarity in the results shows that the analysis is not susceptible to the exact location of windows. When controlling for GC content in correlations between zebra finch/chicken and the ancestral bird linage, we used the average GC content of both chicken and zebra finch as an estimate of GC content in the ancestral lineage.

### Identification of candidate genes for adaptive evolution

#### Rapidly evolving bird (REB) genes

We used a likelihood ratio test to identify genes evolving significantly faster than the average of all genes in a particular lineage. To do so, we compared the likelihood of a model where ω was estimated for a particular gene under consideration, to a null model where ω was fixed to the genome-wide estimate of ω (degrees of freedom (d.f.) = 1), followed by multiple testing correction by false discovery rate (*q *< 0.05) using the program Qvalue [117]. This gives a list of genes that show significantly different ω values, both higher and lower, than the genomic average, of which we considered the genes with higher ω values to represent faster evolving genes.

#### Genes more rapidly evolving in birds (MREB) than in other amniotes

We used the branch model in codeml to identify genes that have evolved significantly faster in a particular lineage compared to the rest of the tree. The null hypothesis assumed that all branches of the tree have the same ω while the alternative hypothesis allows the tested branch to have a different ω. We used a likelihood ratio test with d.f. = 1 to compare the two hypotheses, followed by multiple testing correction by false discovery rate (*q *< 0.05) using Qvalue [117]. This gives a list of genes where ω in the lineage of interest (zebra finch, chicken or ancestral bird lineage) is significantly different, either higher or lower, from ω in the other lineages. We only report the genes that have significantly higher ω values.

#### Genes evolving under positive selection

To detect genes containing codons (at least one) evolving under positive selection in a specific branch (the foreground branch) we used a branch-site test for positive selection [118,119] implemented in the codeml program of the PAML4.1 package. We used the likelihood ratio test 2, with d.f. = 1, with the null hypothesis that ω_2 _was fixed to 1 compared to an alternative model where ω > 1 [120], followed by multiple testing correction by false discovery rate (*q *< 0.05) using Qvalue [117]. For the analysis of positively selected genes, alignments with fewer than 45 codons were excluded. This analysis was based on 8,260 genes in birds and 7,690 genes in eutherian mammals.

### Gene Ontology analysis

To test for overrepresentation of biological processes, molecular functions and cellular components among positively selected or rapidly evolving genes, we performed GO analysis using GoStat [121]. We downloaded GO annotations for chicken, human and mouse from Biomart. The analysis was based on Fisher's exact test between two lists of genes, that is, PS genes and a reference list of all analyzed genes. Multiple testing corrected significance values were based on Benjamini and Hochberg [122] correction (adjusted *P *< 0.1), included with the GoStat software.

### Analysis of neurological genes

The OMIM database [123] was searched on 29 March 2009, using three different search phrases and a search limit set for 'prefix star' (that is, to find only OMIM terms associated with a known gene sequence). One search was on the term 'learning'. To search for genes related to neurogenesis, we used this phrase: [(stem cell AND neur*) OR neurogen*]. To search for genes related to neurodegeneration, we used 'neurogen*'. Human gene IDs in OMIM were cross-referenced and corrected or completed as needed against the HGNC database, and used to retrieve Ensembl gene IDs for human and zebra finch orthologs (Ensembl 53) via Biomart.

## Abbreviations

cM: centiMorgan; d.f.: degrees of freedom; GO: Gene Ontology; MREB: more rapidly evolving in birds; MYA: million years ago; NMDA: N-methyl-D-aspartate; ω: ratio of non-synonymous divergence over synonymous divergence; OMIM: Online Mendelian Inheritance in Man; PS: positive selection; REB: rapidly evolving bird genes.

## Authors' contributions

KN carried out the bioinformatic analyses. KN, AH, CPP and BN participated in the substitution rate analyses. KN, BN and JBWW participated in the bioinformatic analyses of positively selected genes. KN, CM, HS and JBWW participated in the GO analyses. KN, CNB and DFC participated in the analyses of the neurological genes. KN, BN, JBWW and AK participated in the analyses linking recombination rate and efficiency of selection. DFC and HE conceived and designed the study. KN and HE drafted the manuscript. All authors read and approved the final manuscript.

## Supplementary Material

Additional file 1 **Supplementary results**. List of genes corresponding to over-represented GO terms in REB, MREB and PS genes in the different avian lineages. Number of genes identified as positively selected in mammals or as evolving faster in mammals than in other lineages of the amniotes. List of positively selected genes in zebra finch lineage whose human orthologs have been implicated in neurological function (learning, neurogeneration, neurodegeneration). Rate of divergence at fourfold degenerate sites (×10^-9 ^site^-1 ^year^-1^) in Eutherinan lineages. Phylogenetic tree showing the relationship among the species used in the study. Venn diagrams showing for zebra finch, chicken and the ancestral bird lineage the number of REB, MREB, and PS genes. Click here for file

Additional file 2 **Supplementary methods**. Method used to estimate genome-scale ω values. Click here for file
