# Supplementary material for: Molecular evolution of genes in avian genomes
Source: Genome Biol. 2010 Jun 23;11(6):R68. doi: 10.1186/gb-2010-11-6-r68 (PMC2911116; doi:10.1186/gb-2010-11-6-r68)
Supplement: Additional file 1 — Supplementary results. List of genes corresponding to over-represented GO terms in REB, MREB and PS genes in the different avian lineages. Number of genes identified as positively selected in mammals or as evolving faster in mammals than in other lineages of the amniotes. List of positively selected genes in zebra finch lineage whose human orthologs have been implicated in neurological function (learning, neurogeneration, neurodegeneration). Rate of divergence at fourfold degenerate sites (×10-9 site-1 year-1) in Eutherinan lineages. Phylogenetic tree showing the relationship among the species used in the study. Venn diagrams showing for zebra finch, chicken and the ancestral bird lineage the number of REB, MREB, and PS genes. [file gb-2010-11-6-r68-S1.DOC]

Supplementary Table 1. Genes corresponding to over-represented GO terms in RE, MREB and PS genes in the different avian lineages.

| Category | GO | Ensembl ID | Gene |
| --- | --- | --- | --- |
|  | | | |
| **Rapidly evolving (REB) in ancestral bird lineage** | | | |
|  | *Cell adhesion (biological process level 3)* | | |
|  |  | ENSGALG00000006725 | Aggrecan core protein Precursor (Cartilage-specific proteoglycan core protein) |
|  |  | ENSGALG00000011531 | Apoptotic peptidase activating factor 1 |
|  |  | ENSGALG00000005272 | Cadherin 5, type 2, VE-cadherin |
|  |  | ENSGALG00000008109 | Cadherin* |
|  |  | ENSGALG00000007849 | CD44-like protein |
|  |  | ENSGALG00000014582 | Cell adhesion molecule 1 |
|  |  | ENSGALG00000005974 | Collagen alpha-1(VI) chain precursor |
|  |  | ENSGALG00000006958 | Collagen alpha-1(XXVII) chain Precursor |
|  |  | ENSGALG00000003923 | Collagen alpha-3(VI) chain precursor |
|  |  | ENSGALG00000005797 | Collagen, type XX, alpha 1 |
|  |  | ENSGALG00000009523 | Contactin-1 Precursor |
|  |  | ENSGALG00000005426 | FRAS1-related extracellular matrix protein 1 precursor |
|  |  | ENSGALG00000003500 | Hamartin (Tuberous sclerosis 1 protein) |
|  |  | ENSGALG00000009362 | Integrin alpha-6 precursor |
|  |  | ENSGALG00000002389 | Integrin beta-4 precursor |
|  |  | ENSGALG00000011206 | Kit ligand Precursor |
|  |  | ENSGALG00000007113 | Tenascin precursor (TN) (Tenascin-C) |
|  | *Cytokine* *secretion (biological process level 7)* | | |
|  |  | ENSGALG00000014582 | Cell adhesion molecule 1 |
|  |  | ENSGALG00000006527 | Class-I MHC-restricted T cell associated molecule |
|  |  | ENSGALG00000005959 | Glomulin, FKBP associated protein |
|  |  | ENSGALG00000002113 | Lymphocyte cytosolic protein 2 |
|  | | | |
| **Rapidly evolving (REB) in chicken lineage** | | | |
|  | *Telomere maintenance (biological process level 6)* | | |
|  |  | ENSGALG00000010281 | DNA Helicase RECQ Family member (PTHR13710) * |
|  |  | ENSGALG00000009228 | Poly [ADP-ribose] polymerase 1 (PARP-1) |
|  |  | ENSGALG00000013926 | Protein artemis (DNA cross-link repair 1C protein) |
|  |  | ENSGALG00000006123 | Regulator of telomere elongation helicase 1 |
|  |  | ENSGALG00000000657 | Telomeric repeat-binding factor 2 (TTAGGG repeat-binding factor 2) |
|  | *Cholesterol transport (biological process level 6)* | | |
|  |  | ENSGALG00000009955 | ATP-binding cassette, sub-family G (WHITE), member 5 (sterolin 1) |
|  |  | ENSGALG00000009958 | ATP-binding cassette, sub-family G (WHITE), member 8 (sterolin 2) |
|  |  | ENSGALG00000008439 | CD36 antigen |
|  |  | ENSGALG00000004230 | Lipase |
|  |  | ENSGALG00000003018 | Scavenger receptor class B, member 1 |
|  |  | ENSGALG00000004205 | Sterol O-acyltransferase (acyl-Coenzyme A: cholesterol acyltransferase) 1 |
|  |  |  |  |
| **More rapidly evolving in ancestral bird lineage (MREB) than in other amniotes** | | | |
|  | *Cell adhesion (biological level 3)* | | |
|  |  |  |  |
|  | *Blood vessel development/maturation (biological process level 5)* | | |
|  |  | ENSGALG00000005272 | Cadherin-5 precursor (Vascular endothelial cadherin) |
|  |  | ENSGALG00000003580 | Matrix metalloproteinase-28 precursor |
|  |  |  |  |
|  | *Integrin-mediated signaling pathway (biological process level 6)* | | |
|  |  | ENSGALG00000004212 | Disintegrin and metalloproteinase domain 10 |
|  |  | ENSGALG00000009362 | Integrin alpha-6 precursor |
|  |  | ENSGALG00000002389 | Integrin beta-4 precursor |
|  |  |  |  |
|  | *Proteinaceous extracellular matrix (cellular component level 3)* | | |
|  |  | ENSGALG00000005974 | Collagen alpha-1(VI) chain precursor. |
|  |  | ENSGALG00000003923 | Collagen alpha-3(VI) chain precursor |
|  |  | ENSGALG00000003580 | Matrix metalloproteinase-28 precursor |
|  |  | ENSGALG00000012027 | Spondin-1 Precursor |
|  |  | ENSGALG00000007113 | Tenascin precursor (TN) (Tenascin-C) |
|  |  |  |  |
|  | *Synapse organization and biogenesis (biological process level 5)* | | |
|  |  | ENSGALG00000003500 | Hamartin (Tuberous sclerosis 1 protein) |
|  |  | ENSGALG00000000644 | Neurofascin precursor |
|  |  | ENSGALG00000007113 | Tenascin precursor (TN) (Tenascin-C) |
|  |  |  |  |
| **More rapidly evolving in zebra finch lineage (MREB) than in other aminotes** | | | |
|  | *Microtubule cytoskeleton (cellular component level 7)* | | |
|  |  | ENSGALG00000006925 | Centrosomal protein of 110 kDa |
|  |  | ENSGALG00000001111 | Ciliary dynein heavy chain 9 |
|  |  | ENSGALG00000007930 | Dual specificity mitogen-activated protein kinase kinase 5 |
|  |  | ENSGALG00000003170 | Dynein, axonemal, heavy chain 10 isoform 1 |
|  |  | ENSGALG00000000540 | Growth arrest-specific 8 |
|  |  | ENSGALG00000001041 | Katanin p80 WD40-containing subunit B1 |
|  |  | ENSGALG00000000628 | Kinesin family member 21B |
|  |  | ENSGALG00000010057 | Kinesin_motor, Prefoldin |
|  |  | ENSGALG00000021047 | Kinesin-associated protein 3 |
|  |  | ENSGALG00000012160 | Kinesin-like protein KIF18A |
|  |  | ENSGALG00000006221 | Kinesin-like protein KIF1A (Axonal transporter of synaptic vesicles) |
|  |  | ENSGALG00000014737 | Kinesin-like protein KIF2A |
|  |  | ENSGALG00000010120 | Kinesin-like protein KIF2C (Mitotic centromere-associated kinesin) |
|  |  | ENSGALG00000005517 | Kinesin-like protein KIF9 |
|  |  | ENSGALG00000003438 | Kinetochore protein Nuf2 (Cell division cycle-associated protein 1) |
|  |  | ENSGALG00000004515 | Kinetochore-associated protein 1 |
|  |  | ENSGALG00000006657 | Microtubule-associated protein RP/EB family member 1 |
|  |  | ENSGALG00000011479 | Neural precursor cell expressed, developmentally down-regulated 1 |
|  |  | ENSGALG00000003602 | RPGR-interacting protein 1-like protein (Protein fantom) |
|  |  | ENSGALG00000009854 | Serine/threonine-protein kinase Nek2 |
|  |  | ENSGALG00000006604 | Similar to Serine/threonine-protein kinase Nek2 |
|  |  | ENSGALG00000002319 | Similar to tektin 2 |
|  |  | ENSGALG00000005302 | Spindle assembly abnormal protein 6 homolog |
|  |  | ENSGALG00000001348 | Tektin 3 |
|  |  | ENSGALG00000001204 | Uncharacterized protein |
|  | | | |
| **Positively selected (PS) in ancestral bird lineage** | | | |
|  | *Calcium ion binding (molecular function level 5)* | | |
|  |  | ENSGALG00000005299 | Annexin A7 |
|  |  | ENSGALG00000005272 | Cadherin 5, type 2, VE-cadherin |
|  |  | ENSGALG00000004653 | Cadherin-23 precursor |
|  |  | ENSGALG00000009360 | Calpain-2 catalytic subunit precursor |
|  |  | ENSGALG00000005310 | Calsyntenin-2 precursor |
|  |  | ENSGALG00000002287 | Crumbs homolog 1 precursor |
|  |  | ENSGALG00000009058 | Ectonucleoside triphosphate diphosphohydrolase 2 |
|  |  | ENSGALG00000005426 | FRAS1-related extracellular matrix protein 1 precursor |
|  |  | ENSGALG00000004624 | Myosin-5A |
|  |  | ENSGALG00000001314 | Prostaglandin G/H synthase 1 precursor |
|  |  | ENSGALG00000003285 | Protocadherin LKC precursor |
|  |  | ENSGALG00000002744 | Protocadherin-15 |
|  |  | ENSGALG00000007886 | Regeneration associated muscle protease isoform b |
|  |  | ENSGALG00000008456 | Voltage-dependent N-type calcium channel subunit alpha-1B |
|  |  |  |  |
|  | *Homophilic cell adhesion (biological process level 5)* | | |
|  |  | ENSGALG00000005272 | Cadherin 5, type 2, VE-cadherin |
|  |  | ENSGALG00000004653 | Cadherin-23 precursor (Otocadherin) |
|  |  | ENSGALG00000005310 | Calsyntenin-2 precursor |
|  |  | ENSGALG00000003285 | Protocadherin LKC precursor |
|  |  | ENSGALG00000002744 | Protocadherin-15-CD1 |
|  |  |  |  |
| **Positively selected (PS) in chicken lineage** | | | |
|  | *Anion transmembrane transporter activity (molecular function level 6)* | | |
|  |  | ENSGALG00000009061 | Actin-like 6A |
|  |  | ENSGALG00000000820 | 5-hydroxytryptamine (serotonin) receptor 1D |
|  |  | ENSGALG00000007004 | 5-hydroxytryptamine (serotonin) receptor 3A |
|  |  | ENSGALG00000011224 | Anion exchange protein 3 |
|  |  | ENSGALG00000004590 | Chloride channel protein 6 (ClC-6) |
|  |  | ENSGALG00000004134 | Glycine receptor |
|  |  | ENSGALG00000003075 | Na/Pi cotransporter NaPi-IIa |
|  |  | ENSGALG00000008811 | Neutral amino acid transporter A (SATT) (Solute carrier family 1 member 4) |
|  |  | ENSGALG00000004310 | Protein tweety homolog |
|  |  | ENSGALG00000008497 | Sodium-coupled neutral amino acid transporter 2 |
|  |  | ENSGALG00000007018 | Sodium-independent sulfate anion transporter (Solute carrier family 26 member 11) |
|  |  | ENSGALG00000003582 | Solute carrier family 1 member 6 |
|  |  | ENSGALG00000004945 | Solute carrier family 12 (sodium/potassium/chloride transporters), member 1 |
|  |  | ENSGALG00000008874 | Solute carrier family 13 (sodium/sulfate symporters), member 1 |
|  |  | ENSGALG00000004445 | Solute carrier family 13 member 3 |
|  |  | ENSGALG00000010159 | Solute carrier family 20, member 2 |

Supplementary Table 2. Number of genes identified as positively selected in mammals or as evolving faster in mammals than in other lineages of the aminote tree.

|  | Ancestral eutherian lineage | Mouse lineage | Human lineage |
| --- | --- | --- | --- |
| Positively selected^a^ | 98 | 174 | 212 |
| Faster in mammals^b^ | 318 | 340 | 214 |

^a^ Genes with positively selected codons (ω > 1) identified by branch-site model of PAML.

^b^ Genes with higher ω than in avian and lizard branches of the amniote tree, identified by branch model of PAML.

Supplementary Table 3. List of positively selected genes in zebra finch lineage whose human orthologues have been implicated in neurological function (learning, neurogeneration, neurodegeneration).

| ENSEMBL ID | Locus | Gene |
| --- | --- | --- |
| *Learning* |  |  |
| ENSTGUG00000000255 | *DRD2* | D (2) dopamine receptor |
| ENSTGUG00000002630 | *PDE4D* | cAMP-specific 3',5'-cyclic phosphodiesterase 4D |
| ENSTGUG00000003971 | *CREBBP* | CREB-binding protein |
| ENSTGUG00000004191 | *CAMKK1* | Calcium/calmodulin-dependent protein kinase kinase 1 |
| ENSTGUG00000004260 | *PARP1* | Poly [ADP-ribose] polymerase 1 |
| ENSTGUG00000004740 | *PTPRD* | Receptor-type tyrosine-protein phosphatase delta precursor |
| ENSTGUG00000004747 | *GRIN2A* | Glutamate [NMDA] receptor subunit epsilon-1 precursor |
| ENSTGUG00000004856 | *SIM2* | Single-minded homolog 2 |
| ENSTGUG00000005221 | *TSC1* | Hamartin |
| ENSTGUG00000008137 | *VEGFA* | Vascular endothelial growth factor A precursor |
| ENSTGUG00000008638 | *UCHL1* | Ubiquitin carboxyl-terminal hydrolase isozyme L1 |
| ENSTGUG00000010003 | *NPAS2* | Neuronal PAS domain-containing protein 2 |
| ENSTGUG00000010757 | *GRM1* | Metabotropic glutamate receptor 1 precursor |
| ENSTGUG00000012695 | *RIMS1* | Regulating synaptic membrane exocytosis protein 1 |
| ENSTGUG00000017194 | *EPHB2* | Ephrin type-B receptor 2 precursor |
|  |  |  |
| *Neurogenesis* |  |  |
| ENSTGUG00000000120 | *GNE* | Bifunctional UDP-N-acetylglucosamine 2-epimerase/N-acetylmannosamine kinase |
| ENSTGUG00000000275 | *NCAM1* | Neural cell adhesion molecule 1 precursor |
| ENSTGUG00000000589 | *FLI1* | Friend leukemia integration 1 transcription factor |
| ENSTGUG00000000676 | *MEF2C* | Myocyte-specific enhancer factor 2C |
| ENSTGUG00000000694 | *GPR98* | G-protein coupled receptor 98 precursor |
| ENSTGUG00000000847 | *KIRREL3* | Kin of IRRE-like protein 3 precursor |
| ENSTGUG00000002176 | *MCF2* | Proto-oncogene DBL |
| ENSTGUG00000002600 | *STAT3* | Signal transducer and activator of transcription 3 |
| ENSTGUG00000003342 | *TLR4* | Toll-like receptor 4 Precursor |
| ENSTGUG00000003405 | *TOP2B* | DNA topoisomerase 2-beta |
| ENSTGUG00000004013 | *LEF1* | Lymphoid enhancer-binding factor 1 |
| ENSTGUG00000004249 | *ASPM* | Abnormal spindle-like microcephaly-associated protein |
| ENSTGUG00000004260 | *PARP1* | Poly [ADP-ribose] polymerase 1 |
| ENSTGUG00000004464 | *NEFL* | Neurofilament light polypeptide |
| ENSTGUG00000004856 | *SIM2* | Single-minded homolog 2 |
| ENSTGUG00000004881 | *FGFR1* | Basic fibroblast growth factor receptor 1 precursor |
| ENSTGUG00000005467 | *ADIPOQ* | Adiponectin Precursor |
| ENSTGUG00000005684 | *SNRPB* | Small nuclear ribonucleoprotein-associated proteins B and B' |
| ENSTGUG00000005789 | *MYOCD* | Myocardin |
| ENSTGUG00000005986 | *ATXN1* | Ataxin-1 |
| ENSTGUG00000006468 | *AGA* | N(4)-(beta-N-acetylglucosaminyl)-L-asparaginase precursor |
| ENSTGUG00000006519 | *PLP1* | Myelin proteolipid protein |
| ENSTGUG00000006839 | *CACNA1D* | Voltage-dependent L-type calcium channel subunit alpha-1D |
| ENSTGUG00000007050 | *ST6GALNAC* | 5-Alpha-N-acetylgalactosaminide alpha-2,6-sialyltransferase 5 |
| ENSTGUG00000007079 | *LHX2* | LIM/homeobox protein Lhx2 |
| ENSTGUG00000007224 | *PTPRF* | Receptor-type tyrosine-protein phosphatase F precursor |
| ENSTGUG00000008137 | *VEGFA* | Vascular endothelial growth factor A precursor |
| ENSTGUG00000008156 | *POLG* | DNA polymerase subunit gamma-1 |
| ENSTGUG00000008412 | *SEPT5* | Septin-5 |
| ENSTGUG00000010372 | *NOS1* | Nitric oxide synthase, brain |
| ENSTGUG00000011009 | *GFRA1* | GDNF family receptor alpha-1 precursor |
| ENSTGUG00000012615 | *WNK1* | Serine/threonine-protein kinase WNK1 |
| ENSTGUG00000015209 | *SYCP1* | Synaptonemal complex protein 1 |
| ENSTGUG00000017194 | *EPHB2* | Ephrin type-B receptor 2 precursor |
|  |  |  |
| *Neurodegeneration* |  |  |
| ENSTGUG00000003472 | *P4HB* | Protein disulfide-isomerase precursor |
| ENSTGUG00000003971 | *CREBBP* | CREB-binding protein |
| ENSTGUG00000004464 | *NEFL* | Neurofilament light polypeptid |
| ENSTGUG00000004495 | *GAN* | Gigaxonin (Kelch-like protein 16) |
| ENSTGUG00000005484 | *GRIA2* | Glutamate receptor 2 precursor |
| ENSTGUG00000005848 | *PARG* | Poly(ADP-ribose) glycohydrolase |
| ENSTGUG00000005986 | *ATXN1* | Ataxin-1 |
| ENSTGUG00000006468 | *AGA* | N(4)-(beta-N-acetylglucosaminyl)-L-asparaginase precursor |
| ENSTGUG00000007343 | *RAI1* | Retinoic acid-induced protein 1 |
| ENSTGUG00000007440 | *TRPM7-2* | Transient receptor potential cation channel subfamily M member 7 |
| ENSTGUG00000008137 | *VEGFA* | Vascular endothelial growth factor A precursor |
| ENSTGUG00000008156 | *POLG* | DNA polymerase subunit gamma-1 |
| ENSTGUG00000008412 | *SEPT5* | Septin-5 |
| ENSTGUG00000008638 | *UCHL1* | Ubiquitin carboxyl-terminal hydrolase isozyme L1 |
| ENSTGUG00000009490 | *DHCR24* | 24-dehydrocholesterol reductase precursor |
| ENSTGUG00000010221 | *SUMF1* | Sulfatase-modifying factor 1 precursor |
| ENSTGUG00000010372 | *NOS1* | Nitric oxide synthase, brain |
| ENSTGUG00000010433 | *PLA2G6* | 85 kDa calcium-independent phospholipase A2 |
| ENSTGUG00000010918 | *CAPN2* | Calpain 2 |
| ENSTGUG00000012154 | *OSTM1* | Osteopetrosis-associated transmembrane protein 1 precursor |
| ENSTGUG00000012487 | *KLHL1-1* | Kelch-like protein 1 |
| ENSTGUG00000014680 | *HTRA2* | Serine protease HTRA2, mitochondrial precursor |
| ENSTGUG00000018160 | *SLC17A5* | Sialin |

Supplementary Table 4. Rate of divergence at four-fold degenerate sites (x 10^-9^ site^-1^ year^-1^) in Eutherinan lineages. The rates were estimated using divergence times (MYA) corresponding to the mean of the lower and upper bound proposed in Benton and Donoghue (2007).

| Lineage | Divergence | Divergence time | Rate |
| --- | --- | --- | --- |
| Human | 0.148 | 80 | 1.847 |
| Mouse | 0.318 | 80 | 3.977 |
| Ancestral eutherian, after split from marsupials | 0.288 | 150 | 4.120 |

**Supplementary Figures 1-2**

Supplementary Figure 1. Phylogenetic tree showing the relationship among the species used in the study. (a) maximum likelihood tree from 150 randomly chosen protein alignments using the PhyML program, with 100 times of bootstrapping. (b) cladogram indicating the estimated divergence times according to Benton and Donoghue (2007).

a)


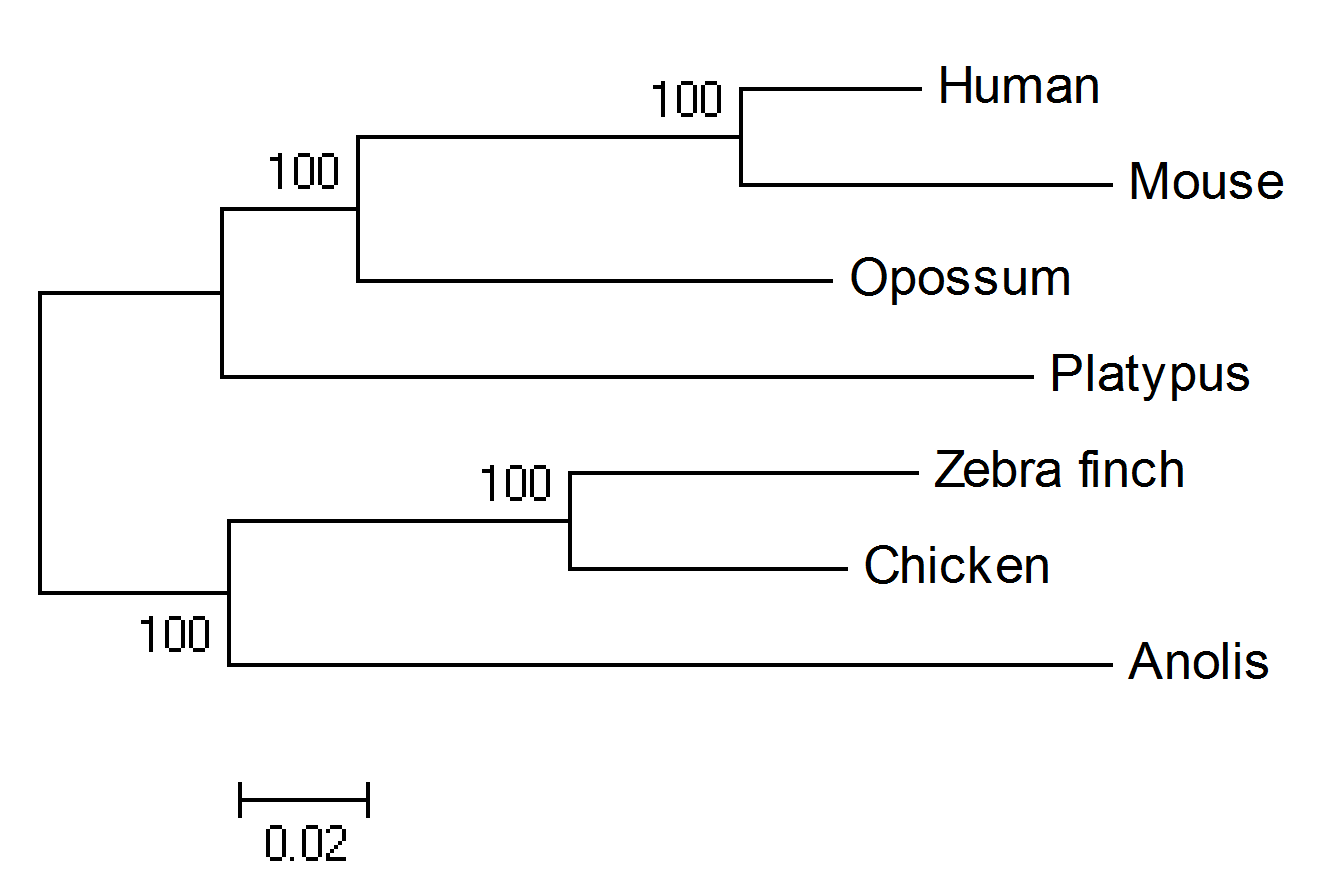


b)


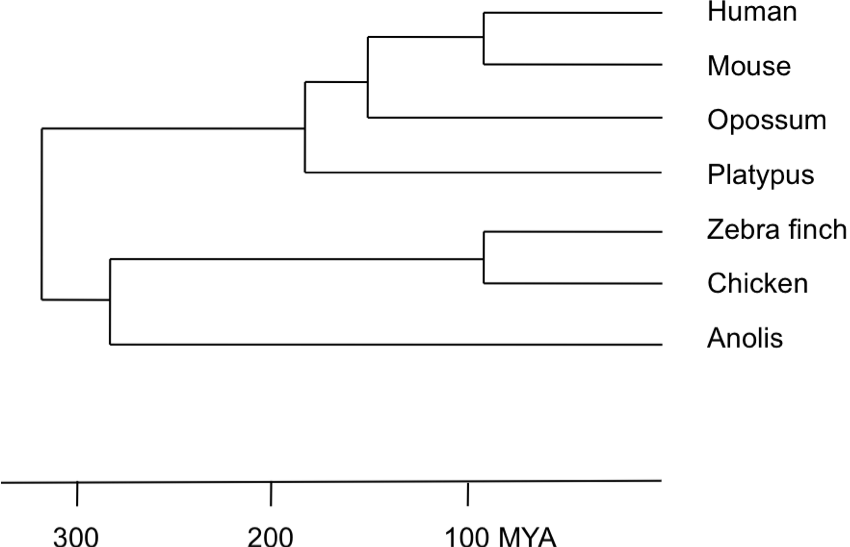


Supplementary Figure 2. Venn diagrams showing for zebra finch, chicken and the ancestral bird lineage the number of (a) genes evolving significantly more rapidly than the genomic average, (b) genes with evidence of positively selected codons and (c) genes evolving faster in avian lineages than in other amniotes.
