# Supplementary material for: Molecular evolution of genes in avian genomes
Source: Genome Biol. 2010 Jun 23;11(6):R68. doi: 10.1186/gb-2010-11-6-r68 (PMC2911116; doi:10.1186/gb-2010-11-6-r68)

**Supplementary Methods**

Estimates of the strength of selection on gene sequences by ω are sensitive to a number of factors, for example, alignment protocol and the model used for estimation of substitution rates. We evaluated the effects of several alternative procedures for estimating ω, as described below.

*Preparation of alignments*

We downloaded protein coding sequence from biomart in ENSEMBL 55 of the chicken (*Gallus gallus*, WASHUC2), zebra finch (*Taeniopygia guttata*, TaeGut3.2.4), green lizard (*Anolis carolinensis*, ANOCAR1), short-tailed opossum (*Monodelphis domestica,* MonDom5), platypus (*Ornithorhynchus anatinus*, OANA5), mouse (*Mus musculus*, NCBIM37) and human (*Homo sapiens*, NCBI36). 1:1 orthologues between zebra finch and chicken were identified using Inparanoid3.0 [112] on basis of a reciprocal Blast best hit approach. We downloaded information on other pairwise orthologous relationships from biomart (www.biomart.org) in ENSEMBL 55, inferring 7,789 1:1:1:1:1:1 orthologs between these six species, followed by making codon-based multiple alignements using MUSCLE3.7 [113].

*Curation of alignemnts*

To test if the MUSCLE protocol provides accurate alignments we estimated ω with and without further curation of the alignments. We estimated overall ω directly from 1,000 alignments where each alignment was made by concatenating 150 randomly chosen alignments, using PAML4.1 [115]. We also used Gblockes [114] to remove poorly aligned codon-positions from these 1,000 concatenated alignments and obtained a new overall ω. Supplementary Methods Table 1 shows that there is a significant difference in the ω estimates in all lineages between the two approaches. ω was consistently higher when not using Gblocks, indicating that the number of non-synonymous substitutions is overestimated due to the presence of incorrectly aligned codons.

*The use of concatenated vs. gene-based alignments*

Estimating ω from concatenated alignments might be considered preferable over using mean ω (or *d_N_*/*d_S_*) from alignments of individual genes as the former show reduced sampling variance due to a higher number of informative sites. However, it can also be argued that estimates from individual genes are more reliable because *d_N_*, *d_S_*, and ω are dependent on other genomic features, such as kappa (the transition/transversion ratio), codon frequency and branch length. Along this way of reasoning it should be beneficial to optimize these parameters for each gene to obtain a more reliable *d_N_*/*d_S_* summary statistic for the genome as a whole. To evaluate this, we estimated mean *d_N_*/*d_S_* (or ω) from 150 randomly chosen alignments in two different ways.

First, we concatenated all the 150 alignments. The overall ω, *d_N_*, and *d_S_* values for the zebra finch lineage were calculated with the two-ratio model in PAML, holding the zebra finch branch as foreground. Secondly, we estimated mean *d_N_* and *d_S_*, from the same 150 alignments using the formula of equation 1 and 2.

(equation 1)
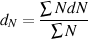


(equation 2)
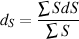


and obtained *d_N_*/*d_S_* simply by dividing mean *d_N_* with mean *d_S_*. Tree length and kappa values were in this case optimized for each alignment.

The estimated overall ω value from the concatenated alignment was 0.1276 (Supplementary Methods Table 2). This is considerably higher than the mean *d_N_*/*d_S_* obtained when using equation 1 and 2, both when optimizing the kappa and branch length values for each gene (0.0919) and when using fixed kappa and branch length values from the concatenated alignment (0.0980). The main reason for a difference between ω from the concatenated alignment and mean *d_N_*/*d_S_* when optimizing kappa and branch length for individual genes is that d_S_ of the latter is much higher (0.2935 vs. 0.2052). This difference is reduced if we only include alignments longer than 1,000 bp (Supplementary Methods Table 2). Shorter alignments thus tend to inflate *d_S_* and result in lower ω values.

*The effect of alignment length*

Among the 8,384 genes used in this study, there were 466, 168 and 214 for which PAML estimated *d_S_* ≤ 0.001 in the ancestral bird, zebra finch and chicken lineages, respectively. Given the considerable length of the branches analyzed, it is unlikely that these genes have had no synonymous changes and that PAML thus underestimated *d_S_*. To test this, we estimated the divergence at third codon positions in the zebra finch lineage using baseml program in PAML. We obtained data for 159 out of 168 alignments, and none of these had a divergence equal to zero; in fact, their divergence was not significantly different to that of all other genes (*p* = 0.274, Wilcoxon rank sum test). However, the mean alignment length of genes with an estimated *d_S_* ≤ 0.001 (384 bp) was significantly shorter than the mean length for all alignments (1307 bp, *p* < 2.2 x 10^-16^), indicating that PAML can underestimate *d_S_* at short alignment lengths. Moreover, the extensive sampling variance associated with rate estimates from short alignments also means that *d_S_* can be overestimated. There was a clear negative relationship between alignment length and *d_S_* (Supplementary Methods Figure 1), which is likely to derive from the fact that when variance is high, *d_S_* estimates can sometimes reach infinity whilst they cannot be lower than 0.

*Free ratio model vs two-ratio model*

We finally tested if the choice of model for d_N_/d_S_ estimation can affect ω estimates. First, in a two-ratio model ω was calculated by specifying one branch of interest as the “foreground branch” while the other branches were deemed to be “background”. This method has the advantage of using a lower number of parameters, but requires an assumption that ω is the same across all “background” branches. Second, we used the parameter-rich free-ratio model, which allows ω to vary across all branches. We found no significant difference in ω values estimated by these two models (*p* = 0.95 in zebra finch lineage, *p* = 1 in chicken lineage, Wilcoxon Rank Sum test). The strength of correlation between ω estimated by the two methods increased with alignment length (Supplementary Methods Table 3).

*Conclusions*

The analyses presented above suggest that the inclusion of poorly aligned sequences will inflate ω estimates. They also indicate that the use of gene-based alignments from which mean ω (or d_N_/d_S_) is estimated tends to inflate d_S_, resulting in underestimation of ω. This is of particular concern for short alignments. Therefore, we chose to use concatenated alignments for estimating overall ω (or d_N_/d_S_), as suggested by Heger & Ponting [25].

Supplementary Methods Table 1. Estimates of mean ω from 1,000 concatenated alignments made by 150 randomly chosen gene-based alignments, with or without using Gblocks for removal of poorly aligned codons. The values in parentheses are 95% confidence intervals based on resampling.

| Gblocks | Pairwise | Zebra Finch | Chicken | Ancestral Bird |
| --- | --- | --- | --- | --- |
| With | 0.1517  (0.1270-0.1788) | 0.1326  (0.1080-0.1601) | 0.1208  (0.0973-0.1527) | 0.1107  (0.0942-0.1295) |
| Without | 0.1575  (0.1308-0.1849) | 0.1422  (0.1140-0.1737) | 0.1340  (0.1051-0.1660) | 0.1158  (0.0926-0.1397) |

Supplementary Methods Table 2. Mean d_N_/d_S_, d_N_ and d_S_ from concatenated or gene-based alignments, in the latter case using either fixed kappa and branch lengths or optimizing these parameters for each gene.

| Method | Length criterion (bp) | d_N_/d_S_ or ω | d_N_ | d_S_ | kappa |
| --- | --- | --- | --- | --- | --- |
| Concatenated | None | 0.1276 | 0.0262 | 0.2052 | 2.05526 |
| Optimized kappa and branch length | None | 0.0919 | 0.0270 | 0.2935 | variable |
| Optimized kappa and branch length | 1000 | 0.1112 | 0.0256 | 0.2305 | variable |
| Fixed kappa and branch length | None | 0.0980 | 0.0221 | 0.2250 | 2.05526 |
| Fixed kappa and branch length | 1000 | 0.0946 | 0.0214 | 0.2264 | 2.05526 |

Supplementary Methods Table 3. The correlation between estimated ω values using the free-ratio model and the two-ratio model, under different alignment length criteria.

| Minimum Length Criterion (bp) | *r^2^* in zebra finch | *r^2^* in chicken |
| --- | --- | --- |
| None | 0.7668 | 0.8382 |
| 500 | 0.8603 | 0.8450 |
| 1,000 | 0.9319 | 0.9453 |
| 5,000 | 0.9981 | 0.9961 |

Supplementary Methods Figure 1. The relationship between log-transformed alignment length and d_S_ in (a) zebra finch, (b) chicken, and (c) ancestral bird lineage. Red lines are median d_S_ and green lines are non-linear regression curves obtained by smooth-spline. Blue points are d_S_ from 1,000 concatenated alignments made by 150 randomly chosen genes. Data points with d_S_ ≤ 0.001 are not included, and the same number of geens with the highest d_S_ estimates were also excluded.


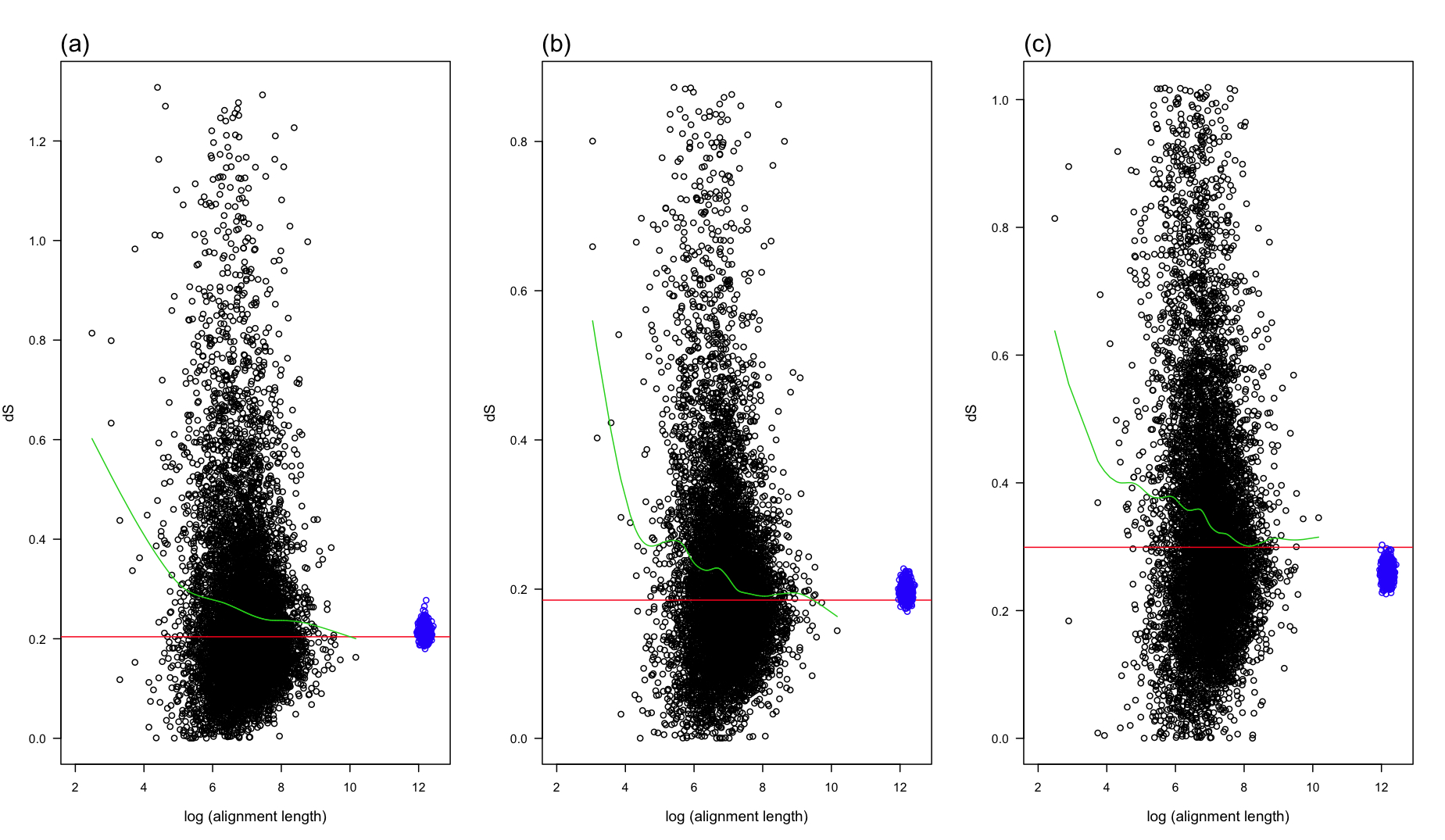

Supplement: Additional file 2 — Supplementary methods. Method used to estimate genome-scale ω values. [file gb-2010-11-6-r68-S2.DOC]
